# A study on the relationship between internet use and cognitive functioning of older adults under the perspective of smart aging

**DOI:** 10.3389/fpubh.2025.1546929

**Published:** 2025-07-07

**Authors:** Jialiang Liu, Yijie Wang, Qiong Wu, Xiaohan Liu, Bin Hu

**Affiliations:** ^1^School of Management, Xuzhou Medical University, Xuzhou, China; ^2^School of Public Health, Xuzhou Medical University, Xuzhou, China

**Keywords:** internet, older adults, cognitive function, influencing factors, propensity to match score

## Abstract

**Background:**

To understand the current status of Internet use among older adults in China and the impact of Internet use on the cognitive function status of older adults.

**Methods:**

Using data from the China Health and Retirement Longitudinal Study (CHARLS) 2020, older adults aged ≥60 years and above were selected. Multiple linear regression was used to analyze the factors affecting Internet use on cognitive function of older adults, propensity matching score (PSM) kernel matching method was used to test the effect of Internet use on cognitive function of older adults, and two methods, proximity matching and radius matching, were used to validate the robustness of the study results.

**Results:**

A total of 5,987 older adults were selected in this study, with cognitive function scores clustered from 9.50 to 14.50. The results of multiple linear regression showed that Internet, age, literacy, home address, self-rated health, life satisfaction, IADL, depression, alcohol consumption, and social participation had an impact on cognitive function of older adults (*p* < 0.05). Among them, Internet use, high literacy, alcohol consumption, and social participation were protective factors for cognitive function in older adults. Age, living in rural areas, IADL disability, and depression are risk factors for cognitive function in older adults.

**Conclusion:**

Cognitive function in older adults is influenced by individual characteristics, health status, and healthy lifestyles. The use of the Internet is conducive to enhancing cognitive function, promoting physical and mental health, and further realizing healthy aging in older adults.

## 1 Introduction

The process of population aging is accelerating, and the proportion of the older adult population continues to rise. According to the seventh national census, by the end of 2020, China’s older population aged 60 years and above accounted for 18.70% of the national population, of which the older adult population aged 65 years and above accounted for 13.50% of the total population (1). Along with the continuous growth of the older population, the incidence of cognitive dysfunction and dementia in the older adult is also on the rise. As the global aging trend is becoming more and more significant, the quality of life and health and well-being of the older adult have become the focus of social attention. The concept of “smart aging” has emerged in recent years (2), which emphasizes the ability of older adults to maintain good physical and mental health and social participation in an active, autonomous and efficient manner with the help of various resources during the aging process. With the rapid development of information technology, the Internet has been deeply integrated into all aspects of social life, and the lifestyles of the older adult have also undergone profound changes. Previous studies on the factors affecting cognitive function in older adults have mostly focused on traditional lifestyles (3), such as diet (4–6), exercise (7, 8), and social activities (9, 10), while the Internet, an emerging and influential factor, has not been sufficiently studied. The Lancet study confirmed nine potentially influential factors that can increase the risk of dementia in older adults, including: low literacy, hypertension, hearing impairment, smoking, obesity, depression, lack of physical activity, diabetes, and low social contact (11). Internet use has been shown to have an improving effect on the health of older adults (12–14), and digitalization can significantly increase life satisfaction among younger older adults (15). Smart aging covers multi-dimensional contents such as the adaptability of the older adult to environmental changes, the rationality of resource utilization, and the continuity of self-development. As a powerful technological tool, the process of using the Internet is rich in “wisdom” features. For example, in terms of adaptive technological design, many Internet applications have developed large font displays, high-contrast interfaces, voice command operations, and simplified interaction processes in response to the physiological characteristics (e.g., declining eyesight and hearing, and reduced hand dexterity) and cognitive limitations (e.g., lower acceptance of complex operations) of the older adult, and these design optimizations have greatly lowered the threshold of the older’s use of the Internet, reflecting the These design optimizations greatly reduce the threshold of the older to use the Internet, reflecting the precise adaptation of technology to the needs of the older, which is a typical manifestation of the older actively adapting to and improving their lives with the help of technology in smart aging (16, 17). The popularization of the Internet provides new opportunities for improving the lives of the older adult, but also brings challenges such as the digital divide (18). An in-depth study of the relationship between Internet use and cognitive functions of the older will help us better understand how to guide the older adult to make reasonable use of the Internet, cross the digital barrier, and realize smart aging, and then provide a scientific basis for the formulation of targeted policies and the development of appropriate digital products and services, so as to promote the construction of an age-friendly society. Therefore, with the help of CHARLS 2020 data, this paper analyzes in depth the impact of Internet use on the cognitive function of the older adult, aiming to seek effective ways to enhance the cognitive function of the older adults.

## 2 Sources and methodology

### 2.1 Data sources

This paper analyzes and researches based on the data from the China Health and Retirement Longitudinal Study (CHARLS) 2020. The CHARLS database is a long-term tracking survey, which targets the middle-aged and older adult population aged 45 years or above, and includes six major parts of the survey: basic information of the individual, household information, health status and functioning, work and retirement, income and expenditure, and epidemiological situation. The current round of CHARLS survey has been approved by the Biomedical Ethics Committee of Peking University under the approval number: IRB00001052-11015.

### 2.2 Subjects of the study

In this study, older people who use the Internet and whose age is greater than or equal to 60 years old were selected. After eliminating the missing values present in the study variables, 5,987 older people who met the requirements were finally selected as the subjects of this study.

### 2.3 Selection of variables

#### 2.3.1 Explained variables

Cognitive functions. In this study, cognitive function was adopted from the Mini-mental State Examination (MMSE) scale, cognitive function mainly includes: situational memory ability (immediate recall ability, delayed recall ability), and mental state (orientation cognitive ability test, drawing ability test, and computational ability test), which was borrowed from existing studies (19, 20). The scale is currently a commonly used international instrument for assessing cognitive functioning, with good reliability and validity (20). Cognitive function scores range from 0 to 21, with larger scores indicating better cognitive function.

#### 2.3.2 Explanatory variables

Internet Use. The specific question in the 2020 CHARLS questionnaire was “In the past month, did you access the Internet?” The specific way of assigning the variable is detailed in Table 1. One was assigned to the older adults who use the Internet and 0 to those who do not use the Internet, referring to the existing study (21).

**Table 1:** Variable assignment.

Variable name	Description of the assignment
Age	1 = 60 ~ 69 2 = 70 ~ 79 3 > =80
Gender	1 = Male 2 = Female
Marital	0 = unmarried 1 = married 2 = divorced 3 = separated 4 = widowed
Educational attainment	0 = illiterate 1 = elementary school 2 = middle school 3 = high school 4 = college and above
Home address	1 = urban 2 = rural
Self-assessment of health	1 = Bad 2 = Fair 3 = Good
ADL	0 = Good 1 = Disabled
IADL	0 = Good 1 = Disabled
Internet	0 = No 1 = Yes
cognitive function	0 to 21 points, the higher the score, the better the cognitive functioning (continuous variable)
Life satisfaction	1 = not at all satisfied 2 = not very satisfied 3 = quite satisfied 4 = very satisfied 5 = extremely satisfied
Depression (CES-D)	0 = No 1 = Yes
Social participation	0 = No 1 = Yes
Drinking wine	0 = No 1 = Yes

#### 2.3.3 Control variables

Individual characteristics, health status and healthy lifestyle. Including age, gender, literacy, marital status, place of residence, self-rated health status, activities of daily living (ADL), instrumental activities of daily living (IADL), life satisfaction, depression, social participation, and alcohol consumption. Drawing on existing studies (22). The specific assignments are shown in Table 1.

### 2.4 Statistical methods

The samples were processed and analyzed using Stata 17.0 and SPSS 27.0 statistical software for data processing and analysis. Count data in descriptive statistics analysis were expressed as (frequency, percentage), Mann–Whitney *U* test and Kruskal–Wallis test were used for one-way analysis, and median and interquartile spacing were used to describe non-normally distributed measures. In this study, multiple linear regression was used to analyze in depth the multiple influences of Internet use on cognitive function in older adults. To further validate the actual impact of the Internet on the cognitive function of older adults, the propensity to match score (PSM) kernel matching method was used to examine the specific impact of Internet use on the cognitive function of older adults, and the results of the study were robustly verified using both proximity matching and radius matching methods. *p* < 0.05 was used to indicate that the difference was statistically significant.

## 3 Results

### 3.1 Basic information about the study population

A total of 5,987 older adults were selected for this study. Among them, 3,376 (56.39*%*) were males and 2,611 (43.61*%*) were females; 4,070 (67.98*%*) were aged 60–69 years, 1,676 (27.99*%*) were aged 70–79 years, and 241 (4.03*%*) were above the age group of 80 years; 21 (0.35*%*) were unmarried, 4,923 (82.23%) were married, 54 (0.90%) were divorced, and 21 (0.35*%*) were separated (82.23*%*), 54 divorced older (0.90*%*), 21 separated older (0.35*%*), and 968 widowed older (16.17*%*); the literacy level of the respondents of this survey was mainly concentrated in the elementary school (50.49*%*) and junior high school (20.96*%*) stages; the older adult living in rural areas were over-represented (3,752,62.67*%*); and the older who assessed themselves as having good health 4,453 were in good health. 4,453 (74.38*%*) were in good health; only 1701 older adults used the Internet, accounting for 28.41% of the total number of respondents; 5,487 (91.65%) were satisfied with their lives; 4,567 (76.28%) were in good ADL status; and 4,576 (76.43%) were in good IADL status; 2,846 (47.54%) of older adults with depressive tendencies; 2,237 (37.36%) of older adults who consume alcohol; 2,944 older adults who are socially engaged; and the cognitive function of older adults in this study was centered on a score of 12.50 (9.50–14.50). Age, gender, marriage, education, address, self-rated health, Internet use, life satisfaction, ADL, IADL, depression, alcohol consumption, and social participation all had an effect on the cognitive function of the older (*p* < 0.05) (see Table 2).

**Table 2:** Descriptive statistical analysis.

Variable name	Number of people	Percentage (*%*)/Median (*P*25, *P*75)	*P*-value
Age (years)	5,987	100	0.000
60 ~ 69	4,070	67.98	–
70 ~ 79	1,676	27.99	–
80 and over	241	4.03	–
Gender	5,987	100	0.000
Male	3,376	56.39	–
Female	2,611	43.61	–
Marital	5,987	100	0.000
Unmarried	21	0.35	–
Married	4,923	82.23	–
Divorcee	54	0.90	–
Separated	21	0.35	–
Widowed	968	16.17	–
Educational attainment	5,987	100	0.000
Illiteracy	948	15.83	–
Primary school	3,023	50.49	–
Middle school	1,255	20.96	–
High school	658	10.99	–
College and above	103	172	–
Home address	5,987	100	0.000
Urban	2,235	37.33	–
Rural	3,752	62.67	–
Self-assessment of health	5,987	100	0.000
Bad	1,534	25.62	–
Fair	3,192	53.32	–
Good	1,261	21.06	–
Internet	5,987	100	0.000
Non-use	4,286	71.59	–
Utilization	1701	28.41	–
Life satisfaction	5,987	100	0.001
Not at all satisfied	122	2.04	–
Not very satisfied	378	6.31	–
Quite satisfied	3,373	56.34	–
Very satisfied	1876	31.33	–
Extremely satisfied	238	3.98	–
ADL	5,987	100	0.000
Good	4,567	76.28	–
Disabled	1,420	23.72	–
IADL	5,987	100	0.000
Good	4,576	76.43	–
Disabled	1,411	23.57	–
Depression	5,987	100	0.000
No	3,141	52.46	–
Yes	2,846	47.54	–
Drinking wine	5,987	100	0.000
No	3,750	62.64	–
Yes	2,237	37.36	–
Social participation	5,987	100	0.000
No	3,043	50.83	–
Yes	2,944	49.17	–
Cognitive function	5,987	12.50 (9.50,14.50)	–

### 3.2 Analysis of the impact of internet use on cognitive functioning in older adults

Cognitive function was used as the dependent variable in the multiple linear regression analysis. Cognitive function scores ranged from 0 to 21, with larger scores indicating better cognitive function in older adults. In order to deeply analyze the effects of Internet use on cognitive function of older adults, the Internet, individual characteristic variables (age, gender, marriage, literacy, home address, and self-assessed health) were included in Model I, and all variables were included in Model II. Internet, age, literacy, home address, and self-assessed health all had an effect on cognitive functioning of older adults in Model I. Internet, age, literacy, home address, self-assessed health, life satisfaction, IADL, depression, alcohol consumption, and social participation were the influencing factors of cognitive functioning of older adults in Model II (*p* < 0.05). It shows that using the Internet, higher literacy, moderate alcohol consumption, and social participation all enhance cognitive function in older adults, and conversely, age, home address, life satisfaction, IADL, and depression are risk factors for cognitive function in older adults (*p* < 0.05) (see Table 3).

**Table 3:** Effects of internet use on cognitive function in older adults.

Variable Name		Model I				Model II			
		β	*95%* confidence interval		*P*-value	β	*95%* confidence interval		*P*-value
Internet	Non-use					0			
	Utilization	1.057	0.874	1.240	<0.001***	0.937	0.756	1.119	<0.001***
Age (years)	60–69 years					0			
	70–79 years	−0.172	−0.344	0.000	0.050**	−0.121	−0.291	0.050	0.165
	80 and over	−0.712	−0.908	−0.516	<0.001***	−0.643	−0.838	−0.449	<0.001***
Gender	Male					0			
	Female	−0.158	−0.320	0.004	0.056*	0.049	−0.125	0.223	0.583
Marital	Unmarried					0			
	Married	1.088	−0.165	2.342	0.089*	1.065	−0.170	2.300	0.091*
	Divorcee	0.662	−0.075	1.400	0.078*	0.685	−0.042	1.412	0.065*
	Separated	−0.295	−0.884	0.294	0.326	−0.195	−0.776	0.385	0.509
	Widowed	0.164	−0.153	0.481	0.310	0.175	−0.137	0.487	0.272
Educational attainment	Illiteracy					0			
	Primary school	2.252	2.030	2.475	<0.001***	2.138	1.918	2.358	<0.001***
	Middle school	1.699	1.565	1.832	<0.001***	1.616	1.484	1.748	<0.001***
	High school	1.340	1.234	1.446	<0.001***	1.264	1.159	1.369	<0.001***
	College and above	1.019	0.863	1.175	<0.001***	0.944	0.790	1.098	<0.001***
Home address	Urban					0			
	Rural	−0.728	−0.893	−0.563	<0.001***	−0.650	−0.813	−0.487	<0.001***
Self-assessment of health	Bad	−0.259	−0.478	−0.040	0.021**	−0.268	0.026	0.509	0.030**
	Fair	−0.030	−0.125	0.066	0.540	0.032	−0.065	0.129	0.516
	Good					0			
Life satisfaction	Not at all satisfied					−0.731	−1.370	−0.092	0.025**
	Not very satisfied					−0.358	−0.595	−0.120	0.003***
	Quite satisfied					−0.002	−0.129	0.125	0.973
	Very satisfied					−0.092	−0.189	0.005	0.064*
	Extremely satisfied					0			
ADL	Good					0			
	Disabled					−0.136	−0.331	0.059	0.172
IADL	Good					0			
	Disabled					−0.577	−0.775	−0.379	<0.001***
Depression	No					0			
	Yes					−0.623	−0.780	−0.467	<0.001***
Drinking wine	No					0			
	Yes					0.271	0.105	0.437	0.001***
Social participation	No					0			
	Yes					0.329	0.180	0.478***	<0.001***

***p < 0.01, **p < 0.05, *p < 0.1.

### 3.3 Endogenous treatment

**Figure 1:**
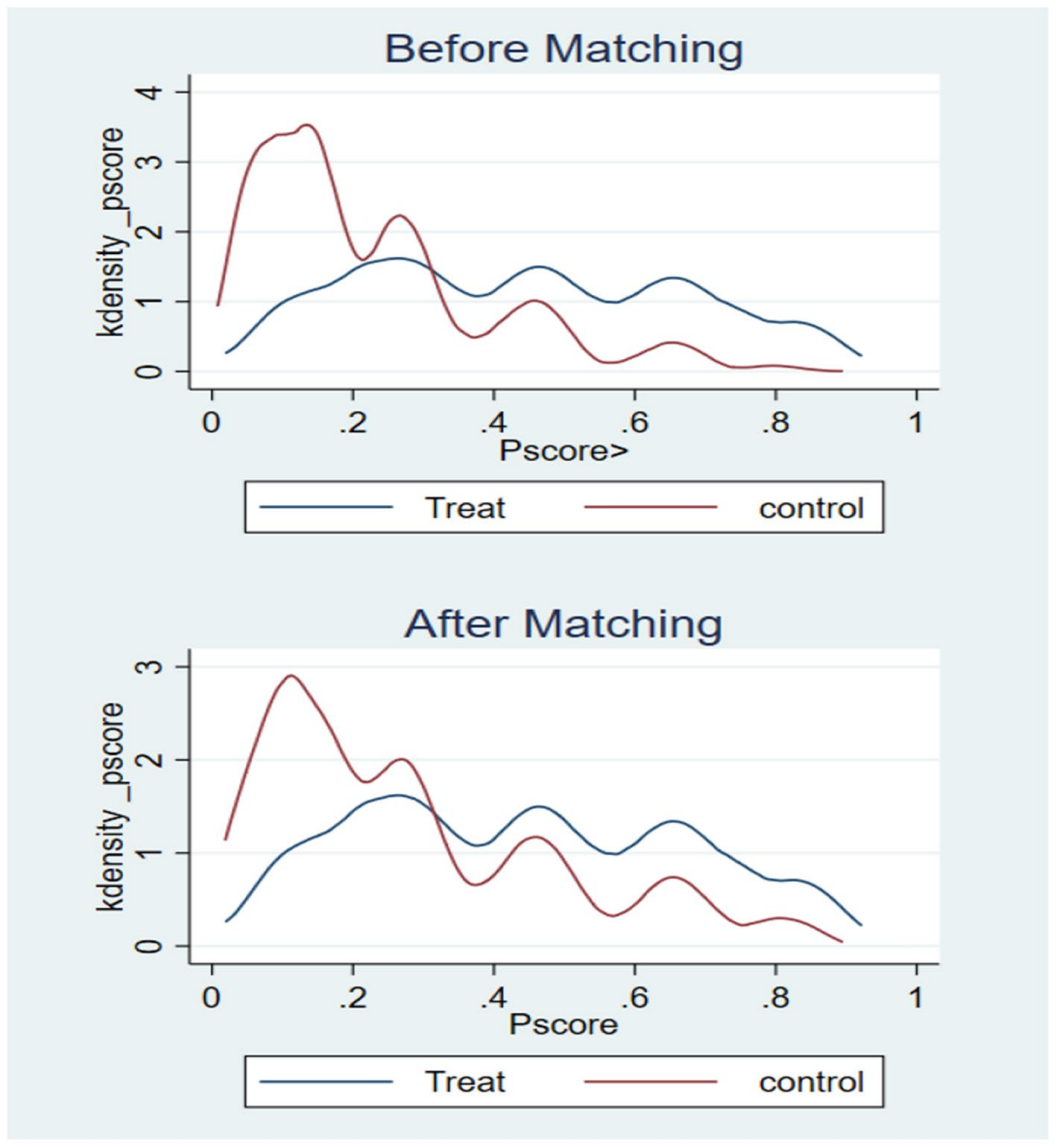
Kernel density matching.

**Figure 2:**
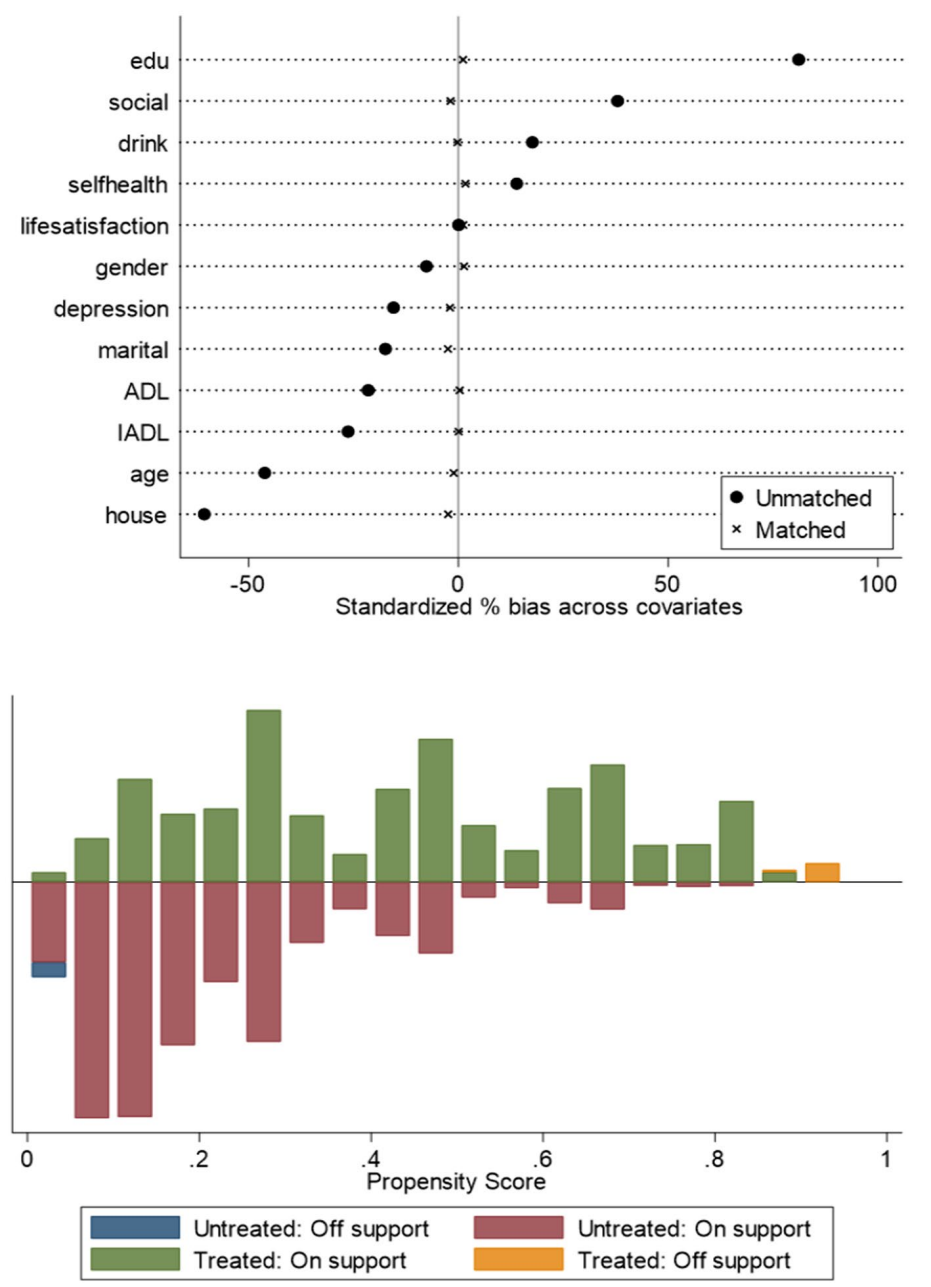
Balance test.

In this paper, older adults who use the Internet are the treatment group and those who do not use the Internet are the control group, and the difference in older adults’ cognitive function between the treatment and control groups is the average treatment effect (ATT) of Internet use on older adults’ cognitive functioning. Analyses were conducted by using kernel matching, which included among the matching variables: age, gender, marriage, literacy, home address, self-rated health, ADL, IADL, depression, alcohol consumption, and social participation. There is a clear separation between the propensity score distributions of the pre-matching treatment group (blue) and the control group (red), while after matching, the distributions of the two groups overlap significantly, indicating that nuclear matching effectively balances the distribution of covariates (see Figure 1). The results of the covariate balance test showed that the standardized bias of each variable after matching was controlled within 10*%* (standardized mean difference *<* 0.1). At the same time, the *t*-test *p*-values of each variable after matching were all greater than 0.05, indicating that there was no significant difference in the distribution of covariates between the treatment group and the control group, and the matching effect was good (see Figure 2 and Table 4). The cognitive function of the treatment group was 13.483 points and that of the control group was 12.474 points, indicating that the cognitive function level of the treatment group was better than that of the control group; the standard error was 0.116, which indicated that the estimation precision of the kernel matching was high. The ATT value was 1.009, and the use of the Internet played a positive impact on the cognitive function of the older adults. The t-value was 8.72, which indicated that there was a significant difference between the treatment and the control groups. Control group are significantly different from each other. In addition, by controlling for confounders and reducing bias using PSM analysis, the estimates of the propensity to match scores were found to be higher compared to the results of the multiple linear regression. This finding highlights the more significant effect of improvement in cognitive functioning in older adults after using the Internet (see Table 5).

**Table 4:** Propensity matching score balance test.

Variable name	Match before/after	Treatment group mean	Control group mean	Deviation (*%*)	*t*-value	*P*-value
Age	Prematch	1.191	1.428	−46.20	−15.08	0.000
	After matching	1.194	1.200	−1.10	−0.39	0.695
Gender	Prematch	1.409	1.447	−7.60	−2.56	0.008
	After matching	1.411	1.405	1.30	0.39	0.697
Marital	Prematch	1.365	1.550	−17.40	−5.86	0.000
	After matching	1.368	1.395	−2.50	−0.78	0.435
Educational attainment	Prematch	1.849	1.114	81.10	29.65	0.000
	After matching	1.813	1.803	1.10	0.30	0.766
Home address	Prematch	1.421	1.708	−60.60	−21.52	0.000
	After matching	1.428	1.440	−2.40	−0.68	0.499
Self-assessment of health	Prematch	2.022	1.928	13.90	4.82	0.000
	After matching	2.016	2.004	1.70	0.49	0.623
Life satisfaction	Prematch	3.289	3.289	0.10	0.02	0.985
	After matching	3.288	3.280	1.10	0.34	0.732
ADL	Prematch	0.174	0.262	−21.50	−7.27	0.000
	After matching	0.176	0.175	0.30	0.09	0.927
IADL	Prematch	0.159	0.266	−26.30	−8.83	0.000
	After matching	0.162	0.162	0.00	0.01	0.989
Depression	Prematch	0.420	0.497	−15.50	−5.38	0.000
	After matching	0.423	0.433	−2.00	−0.58	0.559
Drinking wine	Prematch	0.435	0.349	17.60	6.21	0.000
	After matching	0.428	0.429	−0.20	−0.06	0.950
Social participation	Prematch	0.625	0.439	37.90	13.17	0.000
	After matching	0.619	0.628	−1.90	−0.55	0.581

**Table 5:** Mean treatment effect (ATT) of internet use on the evaluation of cognitive functioning effects in older adults.

Variable name	Methodologies	Process group	Control group	ATT	Standard error	*t*-value
Cognitive function	Kernel matching	13.483	12.474	1.009***	0.116	8.72
	Nearest neighbor matching	13.483	12.686	0.795***	0.143	5.57
	Radius match	13.520	12.476	1.044***	0.136	7.69

***p < 0.001.

### 3.4 Robustness tests

To ensure the robustness of the above results, this paper further validates the impact of Internet use on the cognitive functioning of older adults in China by using propensity to match scores (proximity matching and radius matching). Included in the matching variables are: age, gender, marriage, education level, home address, self-rated health, ADL, IADL, depression, alcohol consumption and social participation. Cognitive function in the near neighbor matching was 13.483 points for the treatment group and 12.686 points for the control group, indicating that older adults who use the Internet have better cognitive function than those who do not. The standard error of 0.143 is slightly larger compared to kernel matching. The ATT value of 0.795 indicates that using the Internet positively contributes to the cognitive functioning of older adults. The *t*-value of 5.57 is smaller compared to kernel matching, but still indicates that the treatment group is still significant in cognitive functioning compared to the control group under the nearest neighbor matching method. Cognitive function in radius matching was 13.520 points for the treatment group and 12.476 points for the control group, which is still higher for the treatment group than the control group. The standard error was 0.136 and the matching accuracy was slightly lower than the kernel matching. The ATT value was 1.044, again indicating that Internet use has a facilitating effect on cognitive functioning of older adults. The t-value was 7.69, again indicating a significant difference between the treatment group and the control group. Taken together, this indicates that all three matching methods show significant differences between the treatment and control groups, which provides strong evidence for further research on the cognitive function of Internet use on older adults (see Table 5).

## 4 Discussion

This paper utilizes the 2020 China Health and Retirement Longitudinal Study (CHARLS) data to deeply analyze the impact of Internet use on the cognitive function of the older adult in China, and the cognitive function scores of the older in this study are mainly clustered in the range of 9.50 to 14.50 points, which is basically in line with the results of Zhu et al. (23).

First, individual characteristics have a significant effect on cognitive functioning of older adults in China. In this study, age, education level, living in rural areas, and poorer self-rated health were found to be influential factors on the cognitive function of older adults. As the age of older adults increases, their cognitive function declines, which is consistent with the results of previous studies (24, 25). In contrast, the use of digitalization enhances the cognitive health of younger and middle-aged older adults (23). It has been found that educational attainment has a significant contributory effect on cognitive functioning in older adults. In general, the higher the educational level of older adults, the better their cognitive function performance. Highly educated older adults tend to have a stronger sense of health investment and are more proactive in personal health management (26). Older adults living in urban areas are more likely to have access to digital resources such as health knowledge and social activities through the Internet, while the digital divide in rural areas is significant, and the lack of technological access may exacerbate the risk of cognitive decline, which suggests that smart aging needs to focus on the coverage of digital infrastructure and aging-appropriate training in rural areas (27). In contrast, the older living in rural areas generally have low literacy, and rural areas are seriously empty-nested, and the older lack opportunities for social interaction with the outside world. The singularity of the life of the older further reduces the cognitive function of the older (28). As age increases, the physical functions of the older gradually deteriorate. The decline in physical health is often accompanied by cognitive decline, which poses a challenge to the daily life and self-care ability of the older (29).

Secondly, health status and healthy lifestyle have a significant effect on cognitive function in older adults. The present study showed that IADL, depression, and alcohol consumption had an impact on cognitive function in older adults. Both ADL and IADL dimensions were considered in this study, and IADL was negatively correlated with cognitive function, while ADL had no clear correlation with cognitive function, which is consistent with previous studies (30). Previous studies have shown that the loss of instrumental activities of daily living (IADL) has a significant role in cognitive dysfunction in older adults (31). IADL is an advanced skill for independent living in older adults, which includes activities of daily living such as cooking, cleaning, and the use of communication devices, and can indirectly respond to older adults’ physical health status. In contrast, IADL is more sensitive in recognizing the older cognitive functioning status of older adults (32). Numerous studies have confirmed that depression has a significant detrimental effect on cognitive function in older adults. Depression damages neurons through multiple mechanisms, which in turn weakens the reserve capacity of cognitive function in older adults. As age increases, the cognitive function of depressed patients gradually shows a downward trend. Under the influence of depressive symptoms, older adults may be at risk for earlier or more frequent cognitive impairment, a condition that seriously affects the quality of life and health of older adults (31, 33, 34). In this study, alcohol consumption was found to have a facilitating effect on cognitive function in older adults. This result is inconsistent with some scholars (35, 36) and may stem from the fact that most older adults show relatively good habits in alcohol consumption. It was found that older adults who drank moderately had lower odds of memory impairment compared to those who abstained from alcohol (37). Moderate alcohol consumption has an improving effect on cognitive function and enhances memory capacity in older adults (38, 39).

Third, Internet, life satisfaction, and social participation have a significant effect on cognitive functioning in older adults. Moderate Internet use improves cognitive function and reduces the risk of cognitive impairment and depression in older adults (13, 14, 40–42), but overuse of the Internet can lead to cognitive decline in older adults (43). Li et al. (44) study confirms the importance of Internet use in preserving cognitive function in older adults, especially those who live alone, are accompanied by a spouse only, or are part of a two-generation household. During the COVID-19 pandemic, Internet use was associated with significant improvements in cognitive function among Swiss older adults (45). Internet use is not only a form of cognitive stimulation but also an expression of social engagement. The sense of belonging and the formation of social networks created during online social activities further contribute to brain functioning (46). The use of social software requires older adults to repeatedly learn a variety of skills, including: video calling, taking pictures, etc., and this training deepens older adults’ long-term memory capacity (47). It has been shown that the combination of graphic and verbal information has a positive effect on older people’s integration of information in their long-term memory, promoting active cognitive processing in working memory, thus improving the level of attention of older people (48). From the perspective of smart aging, the Internet is not only a cognitive stimulation tool, but also a core means of bridging the digital divide: on the one hand, the older adult receive cognitive training (e.g., memory and attention activation) through the video call and graphic information processing functions of social software (e.g., WeChat), forming the ‘operation – memory reinforcement’ cycle (48). On the other hand, age-appropriate technology design (e.g., large fonts, voice commands) reduces the operational load and makes it easier for low-educated older adults to participate in social activities via the Internet, thus enhancing the ‘social participation – cognitive gain’ effect (49, 50). There is a close link between life satisfaction and cognitive function, and life dissatisfaction may exacerbate the decline of cognitive function, thus further affecting the mental health status of older adults. Enhancing life satisfaction in older adults is important for maintaining cognitive function and promoting healthy aging (51). Social participation has a positive response to cognitive function in older adults, which is consistent with the results of Zhang et al. (52) and Chien et al. (53). Older adults are able to obtain the emotional support and value recognition that older adults need in the process of socializing with friends and family, which promotes their physical and mental health (54). Therefore, focus should be placed on enhancing the social participation of older adults to strengthen their cognitive function and reduce the risk of cognitive impairment, thereby promoting the enjoyment of a healthier and more fulfilling later life.

In summary, Internet use is associated with better cognitive function among older adults and is linked to a reduced risk of cognitive impairment. In addition, special attention needs to be paid to the cognitive functioning of rural older persons, and training in the use of the Internet needs to be widely carried out to improve the mental health of older persons and further enhance their social participation, so as to promote the physical and mental health of older persons.

## 5 Conclusion

This study synthesized and analyzed the cognitive function and its related influencing factors in 5987 older adults. The results showed that cognitive functioning of older adults was significantly influenced by individual characteristics (age, literacy, and residential environment), health status (self-rated health, depression, and IADL), and healthy lifestyles (Internet use, alcohol consumption, and social participation). The multifaceted factors affecting cognitive functioning in older adults were revealed, suggesting that the development of individualized interventions should be emphasized in the process of promoting healthy aging in older adults. By encouraging older adults to use the Internet appropriately, enhancing their cultural literacy, improving their lifestyles, and increasing their social participation, not only can their cognitive functions be effectively enhanced, but also their overall physical and mental health can be promoted, thus providing strong support for the realization of healthy aging. There is also a need to pay special attention to the cognitive functioning of rural older persons, to provide extensive training in the use of the Internet, to improve the mental health of older persons, to further enhance their social participation and to promote their physical and mental health.

## 6 Limitation

There are some limitations to this study. The data used in this paper are cross-sectional, reflecting only a particular point in time and failing to capture trends over time. Future studies could incorporate multi-year data for longitudinal analysis to more comprehensively reveal the impact of Internet use on the cognitive function of older adults in China. In addition, this study did not take into account the differences in Internet use history (new versus long-term users), which may also have some impact on the results.

## Data Availability

The data analyzed in this study is subject to the following licenses/restrictions. Requests to access these datasets should be directed to hubin@xzhmu.edu.cn.
